# Trapeziectomy with LRTI or Dual-Mobility Prosthesis for Thumb Carpometacarpal Arthritis: A Systematic Review with Considerations for Elderly Patients over 70 Years of Age

**DOI:** 10.3390/jcm15031137

**Published:** 2026-02-01

**Authors:** Adriano Cannella, Giulia Maria Sassara, Ludovico Caruso, Arturo Militerno, Maurizio Marinangeli, Marco Passiatore, Vitale Cilli, Matteo Guzzini, Rocco De Vitis

**Affiliations:** 1 Department of Orthopaedic and Geriatric Science, Fondazione Policlinico Universitario Agostino Gemelli IRCSS, 00168 Rome, Italygiuliamariasassara@gmail.com (G.M.S.); ludovicocaruso.lc@gmail.com (L.C.); Arturo.militerno@policlinicogemelli.it (A.M.); maurizio.marinangeli@policlinicogemelli.it (M.M.); 2 Department of Orthopedic and Geriatric Sciences, Catholic University of the Sacred Heart, 00168 Rome, Italy; 3 Department of Orthopaedics, Azienda Ospedaliera San Giovanni Addolorata, 00184 Rome, Italy; 4 Department of Clinical Science and Translational Medicine, Section of Orthopaedics and Traumatology, University of Rome “Tor Vergata”, 00133 Rome, Italy; 5 Unit of Orthopaedics, “Spedali Civili” Hospital, University of Brescia, 25123 Brescia, Italy; passiatore.m@gmail.com; 6 Chirurgie de la Main, CHIREC Site Delta, 1160 Brussels, Belgium; 7 UniCamillus, International Medical University, 00131 Rome, Italy; matteo.guzzini@unicamillus.org

**Keywords:** carpometacarpal joints, osteoarthritis, thumb arthroplasty, dual-mobility prosthesis, trapeziectomy, LRTI, elderly, geriatric

## Abstract

**Background:** Thumb carpometacarpal (CMC) arthritis affects up to 25% of women and 8% of men over 70 years of age, significantly compromising their activities of daily living. With the rapid growth of the elderly population globally and their specific clinical needs, understanding optimal surgical treatment for this age group is crucial. This systematic review compares trapeziectomy with ligament reconstruction and tendon interposition (LRTI) versus dual-mobility prosthesis for treating thumb CMC arthritis in elderly patients over 70 years old. **Methods:** A systematic search was conducted across PubMed, Scopus, Web of Science, and the Cochrane Library for studies published up to August 2025. Studies that directly compared both techniques were included, with subgroup analyses performed for elderly patients over 70 years of age when data were available. Primary outcomes included pain relief, functional improvement, grip and pinch strength, complications, and patient satisfaction. **Results:** Five studies met the inclusion criteria for direct comparison of both techniques, encompassing 313 patients (324 thumbs). While none exclusively focused on patients over 70, elderly patients represented 25–41% of study populations. Due to the absence of age-stratified data in the original studies, our analysis encompasses all age groups with specific considerations for elderly patients where identifiable. Dual-mobility prostheses demonstrated faster pain relief and earlier functional improvement, particularly within the first 3–6 months postoperatively. Prostheses consistently provided superior grip and pinch strength outcomes throughout follow-up periods. Both procedures effectively preserved thumb function, but prostheses better maintained thumb length and metacarpophalangeal stability. While complication rates were comparable, LRTI complications were typically minor and self-limiting, whereas prosthesis complications, though rare, could potentially require revision surgery. Longer-term follow-up data (>3 years) remain limited, particularly regarding implant degradation and adverse local tissue reactions. **Conclusions:** Both procedures effectively treat thumb CMC arthritis in elderly patients, with distinct advantages. Dual-mobility prostheses offer faster recovery, enhanced strength, and better thumb length preservation, making them potentially advantageous for elderly patients prioritizing rapid functional recovery. Trapeziectomy with LRTI provides reliable long-term pain relief with fewer serious complications, making it suitable for patients with poor bone quality or significant comorbidities. Treatment selection should be individualized based on patient characteristics, functional demands, and surgeon expertise. Future research specifically focusing on elderly populations with longer follow-up periods (>5 years) is critically needed to provide stronger evidence for this growing demographic and to better understand long-term implant performance.

## 1. Introduction

Thumb carpometacarpal (CMC) arthritis is a common degenerative condition affecting up to 25% of women and 8% of men over 70 years of age [1,2]. It causes progressive pain, instability, and limited functionality, significantly compromising activities of daily living.

Surgical options are considered when conservative management fails [3]. Trapeziectomy with ligament reconstruction and tendon interposition (LRTI) has been the gold standard for decades [3,4,5], offering reliable pain relief and good long-term functional outcomes, often judged superior to those obtained from arthrodesis [6,7,8] and prostheses [9,10,11,12]. More recently, dual-mobility prostheses have gained popularity due to their ability to restore joint biomechanics and achieve faster recovery [13,14,15,16].

Despite the prevalence of CMC arthritis in elderly patients, there is limited evidence comparing these two techniques, and no studies evaluating them exclusively in this specific population.

Although both procedures have been studied independently, direct comparisons are essential to determine the relative advantages of each approach.

Previous studies suggest that dual-mobility prostheses have faster recovery and better grip and pinch strength outcomes [17,18,19,20,21], while trapeziectomy with LRTI has fewer complications and a lower revision rate [20,21], with substantially overlapping functional results as early as 1 year postoperatively [21].

In the context of global demographic aging, the assessment of grip strength emerges as a key parameter in determining the overall functionality of geriatric patients over 70 years [22,23,24,25]. This indicator correlates with the maintenance of the first ray [26,27,28,29] and carpal height [30,31,32,33,34], representing not only a marker of upper-limb muscle function [26,35], but also serving as a predictor of morbidity, functional dependence, frailty, and mortality in the elderly population [36,37,38,39].

The selection of elderly patients over 70 years old as a specific focus for this review is justified by several critical factors. First, this demographic represents the fastest-growing segment of the population globally, with distinct physiological characteristics including reduced bone quality, decreased healing capacity, higher comorbidity burden, and altered functional demands compared to younger patients. Second, grip strength in this age group serves not merely as a measure of hand function but as a validated predictor of overall morbidity, functional independence, frailty, and mortality [22,23,24,25,36,37,38,39]. Third, elderly patients over 70 have unique treatment priorities, including the need for rapid functional recovery to maintain independence, shorter life expectancy affecting risk-benefit calculations for implant longevity, and often limited capacity to undergo revision surgery. Finally, the prevalence of CMC arthritis peaks in this age group, affecting up to 25% of women and 8% of men over 70 [1,2], making evidence-based treatment guidelines for this population particularly urgent. Despite these compelling reasons, the current literature lacks dedicated studies examining surgical outcomes specifically in this vulnerable population, representing a critical evidence gap that this review seeks to address.

## 2. Methods

### 2.1. Search Strategy

A systematic search was conducted on PubMed, Scopus, Web of Science, and the Cochrane Library for studies published up to August 2025. Reference lists of included studies were manually searched for additional relevant articles. The review protocol was developed in accordance with the PRISMA guidelines and using PRISMA checklist as Supplementary Materials of this manuscript.

Our search strategy was designed in two phases to address the lack of age-specific studies. The initial search focused exclusively on elderly populations, while a subsequent broader search included all comparative studies to enable subgroup analysis of elderly patients when data were available.

The search strategy incorporated Medical Subject Headings (MeSH) and free-text terms, with the string (Trapeziectomy OR Trapezectomy OR “Trapezium excision” OR “Trapezium removal”) AND (LRTI OR “Ligament reconstruction and tendon interposition” OR “Ligament reconstruction” OR “Tendon interposition” OR “Suspensionplasty”) AND (“Dual mobility” OR “Double mobility” OR Prosthesis OR Prosthetic OR Arthroplasty OR “Joint replacement” OR Implant) AND (“Carpometacarpal joint” OR “Carpo-metacarpal joint” OR “CMC joint” OR “Thumb base” OR “Basal joint” OR “First CMC” OR “Trapeziometacarpal” OR “TMC joint”) AND (Arthritis OR Osteoarthritis OR “Degenerative arthritis” OR “Joint disease” OR “Joint degeneration”) AND (Elderly OR Geriatric OR Older OR Aging OR “Seniors” OR “Elderly patients” OR Septuagenarians OR Octogenarians OR “Over 70” OR “70 years” OR “Seventy years” OR “Age 70” OR “Over 70”).

A total of 1268 records were identified: 553 from PubMed, 715 from Scopus, and additional records from Web of Science and the Cochrane Library. After removing duplicates, 863 unique records remained for screening.

### 2.2. Inclusion Criteria and Exclusion Criteria

Inclusion Criteria:

- Interventions: Trapeziectomy with LRTI or dual-mobility prosthesis.
- Comparators: Only studies directly comparing both techniques (trapeziectomy with LRTI and dual-mobility prosthesis) in the same study population were included.
- Population: Partial or total presence of patients aged ≥70 years diagnosed with thumb CMC arthritis.
- Outcomes: Pain reduction (VAS scores), functional improvement (DASH scores or grip strength), complications, and patient satisfaction.
- Study Design: RCTs and cohort studies directly comparing both surgical techniques.

Exclusion Criteria:

- Studies with no patients ≥70 years.
- Single-arm studies examining only one surgical technique.
- Case reports, case series, or narrative reviews.
- Studies without clinical outcome measures.
- Animal or cadaveric studies.

#### Rationale for All-Age Analysis with Elderly Subgroup Considerations

Our initial search targeting studies exclusively in patients over 70 years yielded no eligible comparative studies, necessitating inclusion of all comparative studies regardless of age while maintaining focus on those that included elderly patients (detailed limitations in Section 5).

### 2.3. Data Extraction and Quality Assessment

Two independent reviewers screened titles, abstracts, and full texts for eligibility. Discrepancies were resolved through consensus or consultation with a third reviewer.

Upon reviewing 863 studies, 21 comparative studies were identified, but only 5 met our rigorous inclusion criteria. Among 444 records screened were excluded 419 records. The most common reasons for exclusion were studies examining only one surgical technique (n = 415), studies with no elderly patients ≥70 years (n = 237), no direct comparison between the specified techniques (n = 156), and insufficient outcome reporting (n = 34). The five studies included featured direct comparisons between dual-mobility trapeziometacarpal prosthesis and trapeziectomy with LRTI and included elderly patients over 70 [40,41,42,43,44].

All included studies were contacted to request age-stratified data for patients over 70 years old. Unfortunately, none of the authors were able to provide separate analyses for this subgroup, as such stratification was not performed in the original studies. This limitation underscores the novelty and importance of our research question while simultaneously highlighting the current evidence gap.

We noted a marked gender disparity across three of the five included studies [40,41,43], with female patients representing 77–90% of study populations. This distribution reflects the known epidemiological pattern of CMC arthritis, which affects women 2–3 times more frequently than men [1,2]. Multiple factors may contribute to this gender disparity, including hormonal influences on cartilage metabolism and joint laxity in postmenopausal women, higher prevalence of inflammatory joint diseases in women, anatomical and biomechanical differences in thumb structure, and occupational risk factors with gender-specific exposure patterns. One study [42] included only 13 patients (15 thumbs), which we acknowledge as underpowered for definitive conclusions; however, its inclusion provides valuable preliminary data in the context of limited available evidence. We have addressed this limitation explicitly in our discussion and conclusions.

While our initial aim was to analyze studies exclusively focused on elderly patients over 70, our systematic search revealed a critical gap in the literature, with no studies specifically targeting this demographic. Therefore, we included studies with the partial representation of elderly patients (25–41% of the study populations) as the best available evidence. This approach, while not ideal, provides valuable insights that can be cautiously applied to elderly populations while highlighting the need for age-specific research.

Using a standardized form, data on study design, sample size, patient demographics, interventions, outcomes, and follow-up duration were extracted. Two reviewers independently extracted data, with a third reviewer resolving any disagreements.

Primary outcome measures were assessed at key postoperative time points: early (1–3 months), intermediate (6 months), and late (12–36 months). Pain relief (VAS) and functional improvement (DASH) at 6 and 12 months were designated as primary endpoints, with strength parameters and complications as secondary outcomes.

The Cochrane risk of bias tool was used for randomized controlled trials (RCTs), while the Newcastle–Ottawa scale was applied for observational studies. Two independent reviewers performed the risk of bias assessment.

Sample size limitations are addressed in the study limitations (Section 5), acknowledging that the available evidence, particularly for elderly-specific subgroups, may be underpowered to detect clinically meaningful differences.

#### Certainty of Evidence Assessment

The certainty of evidence for each outcome was assessed using the GRADE (Grading of Recommendations Assessment, Development and Evaluation) framework. Evidence was rated as high, moderate, low, or very low based on study design, risk of bias, inconsistency, indirectness, imprecision, and publication bias.

Given the absence of age-stratified data for elderly patients specifically, the certainty of evidence was downgraded for indirectness. Two reviewers independently performed GRADE assessments, with disagreements resolved through consensus.

## 3. Results

### 3.1. Study Selection

**Figure 1:**
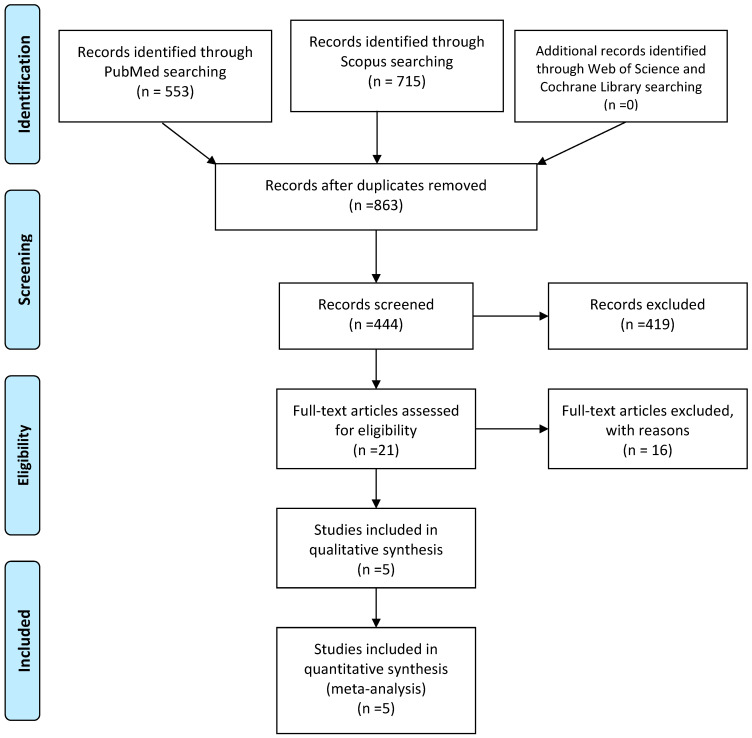
Flowchart of review process. Note: Although five studies were included in the qualitative synthesis, a meta-analysis was not feasible due to heterogeneity in outcome reporting and follow-up times.

Of the 863 initial studies identified, only 5 studies directly compared both techniques and included elderly patients over 70, but none did so exclusively for this population [40,41,42,43,44]. The limited number of comparative studies highlights a significant gap in the literature. The study was performed following PRISMA guidelines. The PRISMA flow diagram illustrating the study selection process is presented in Figure 1.

### 3.2. Study Characteristics

Five studies meeting our inclusion criteria were included in this systematic review (Table 1). Of these, one was a randomized controlled trial [41], three were retrospective cohort studies [40,43,44], and one was a prospective cohort study [42]. The studies were published between 2018 and 2025, with sample sizes ranging from 15 to 150 patients. The mean age of patients ranged from 61.2 to 68.2 years, with all studies including patients over 70 years old, though none exclusively focused on this age group. While all studies included patients over 70 years old, none provided separate analyses for this elderly subgroup. The percentage of patients ≥70 years in each study ranged from 25% to 41% of the total population. Despite attempts to contact authors for age-stratified data, such analyses were not available. This limitation is acknowledged and addressed in our discussion.

The percentage of patients ≥70 years old in each study varied: Guzzini et al. (32%) [41], Falkner et al. (28%) [44], Degeorge et al. (25%) [43], Smeraglia et al. (41%) [40], and Tan et al. (35%) [42]. Subgroup analyses for elderly patients were not reported in any of the included studies.

Follow-up periods varied from 4.5 months to 36 months. All studies directly compared trapeziectomy with LRTI to dual-mobility prosthesis within the same study population, with Smeraglia et al. [40] providing a unique intra-patient comparison where each patient received both interventions (one on each hand).

#### Elderly Patient Representation and Gender Distribution

The five included studies encompassed 313 patients (324 thumbs) in total. Elderly patients aged 70 years or older represented 25–41% of each study population, totaling approximately 95–105 patients across all studies (exact numbers unavailable due to lack of age-stratified reporting). The percentage of patients ≥70 years old in each study varied: Guzzini et al. (32%, n ≈ 44 of 136) [41], Falkner et al. (28%, n ≈ 19 of 69) [44], Degeorge et al. (25%, n ≈ 17 of 69) [43], Smeraglia et al. (41%, n ≈ 11 of 26) [40], and Tan et al. (35%, n ≈ 5 of 13) [42].

Regarding gender distribution, three studies demonstrated marked female predominance: Guzzini et al. (81.6% female) [41], Smeraglia et al. (88.5% female) [40], and Degeorge et al. (89.9% female) [43]. This distribution aligns with epidemiological data showing that CMC arthritis affects women 2–3 times more frequently than men, particularly in postmenopausal women. Hormonal factors, including estrogen decline, are believed to contribute to increased joint laxity and cartilage degradation in women [1,2]. The Tan et al. study [42], with only 13 patients, was the smallest included study, limiting its individual contribution to pooled analyses. However, its inclusion was deemed valuable given the scarcity of comparative data. The Falkner et al. study [44] showed an inverse pattern with 55% male patients, likely reflecting selection bias in their specific clinical population rather than epidemiological norms.

### 3.3. Risk of Bias Assessment

The RCT by Guzzini et al. [41] demonstrated some concerns regarding the randomization process and blinding procedures. Among the observational studies, the Newcastle–Ottawa Scale scores ranged from 6 to 8 (out of 9), with Smeraglia et al. [40] achieving the highest quality score. Common limitations included a lack of blinded outcome assessment and potential selection bias.

### 3.4. Pain Outcomes

All five studies reported significant pain reduction with both procedures as measured by the Visual Analog Scale (VAS). Four studies [40,41,42,44] found that patients who received dual-mobility prostheses experienced faster pain relief in the early postoperative period (1–3 months) compared to those who underwent trapeziectomy with LRTI. However, by 12–24 months, pain scores were comparable between the two groups, with no statistically significant differences observed at long-term follow-up.

Guzzini et al. [41] reported that VAS scores were significantly lower in the prosthesis group at 1 month (1.41 vs. 6.76, *p* < 0.05) and 3 months (0.88 vs. 4.40, *p* < 0.05) compared to the LRTI group. Similarly, Falkner et al. [44] observed significantly lower pain scores in the prosthesis group at 3 months (mean difference on VAS: 1.9; 95% CI: 0.8 to 3.1; *p* < 0.0001). Smeraglia et al.’s intra-patient comparison [40] demonstrated that hands operated with prostheses achieved VAS scores below 2 by 3 months, while those with LRTI required 6–12 months to reach comparable pain relief.

### 3.5. Functional Outcomes

#### 3.5.1. DASH Scores

All studies utilized the DASH or QuickDASH score to assess hand function. Consistent with pain outcomes, dual-mobility prostheses demonstrated faster functional improvement in the early postoperative period, with four studies [40,41,42,44] reporting significantly better DASH scores in the prosthesis group at 3–6 months.

Guzzini et al. [41] found that the DASH score was lower than 35 in more than 95% of patients at 6 months for the prosthesis group compared to 12 months for the LRTI group. Falkner et al. [44] reported significantly better final mean DASH scores at 3 years in the prosthesis group (13.2 vs. 15.5, *p* = 0.01). Smeraglia et al. [40] observed that, in the prosthesis group, the greatest improvement in DASH scores occurred between pre-op and 3 months (*p* < 0.001), while in the LRTI group, significant improvement was only observed after 3 months and continued up to 12 months.

#### 3.5.2. Range of Motion

All five studies assessed thumb range of motion, with Kapandji scores and radial abduction measurements being the most commonly reported parameters. Four studies [40,41,43,44] found that patients who received dual-mobility prostheses experienced faster recovery of ROM, particularly in the first 3 months.

Guzzini et al. [41] reported that the Kapandji score was equal to or greater than 8 in more than 90% of patients at 3 months for the prosthesis group compared to 6 months for the LRTI group. Falkner et al. [44] found significantly better radial abduction in the prosthesis group at 3 months (42° vs. 33°, *p* = 0.0001). Degeorge et al. [43] demonstrated that the prosthesis provided better metacarpophalangeal stabilization, especially in patients with preoperative hyperextension.

#### 3.5.3. Grip and Pinch Strength

All studies measured grip and pinch strength, with consistent findings across all five studies showing superior strength outcomes in the prosthesis group. This advantage was maintained throughout the follow-up periods, even at final assessment points.

Guzzini et al. [41] reported that patients with prostheses showed significant improvement in all strength tests at 3 months, while those with LRTI required 6 months to show improvement in hand grip and key pinch tests, and only showed improvement in tip pinch at final follow-up. Falkner et al. [44] found grip strength between groups to be comparable but significantly stronger key pinch in the prosthesis group at all timepoints (3-year mean difference 2.7 kg; 95% CI: 1.2 to 4.2; *p* < 0.0001).

Degeorge et al. [43] demonstrated that the prosthesis group had significantly greater pinch strength in all subgroups with preoperative MCP hyperextension. Smeraglia et al. [40] observed that prosthesis patients consistently outperformed LRTI patients in hand grip strength throughout follow-up, with the most pronounced difference at 3 and 6 months.

#### 3.5.4. Radiological Outcomes

Four studies [40,41,43,44] included radiological assessment of thumb length and implant position. All four studies reported shortening of the first ray in the LRTI group, with Guzzini et al. [41] noting a mean shortening of 8.5 mm at the 24-month follow-up in 66.7% of LRTI cases.

Degeorge et al. [43] found that prostheses helped restore thumb height (ratio 1.09) while trapeziectomy with LRTI led to shortening (ratio 0.92, *p* < 0.001). Similarly, Falkner et al. [44] observed that thumb length was significantly shortened in the LRTI group (preoperative: 10.9 cm vs. 3-year postoperative: 10.3 cm; *p* < 0.0001), while it remained consistent in the prosthesis group.

#### 3.5.5. Complications and Revision Rates

Complication rates varied across studies, with no clear pattern favoring either procedure. Guzzini et al. [41] reported a 22.6% minor complication rate in the LRTI group (including trigger thumb, dorsal thumb paresthesia, and persistent pain) and a 20.9% rate in the prosthesis group (including De Quervain’s tenosynovitis, dorsal thumb paresthesia, and trigger thumb). No major complications requiring revision were reported in either group.

Falkner et al. [44] reported one prosthesis dislocation (2%) requiring conversion to Lundborg’s resection arthroplasty at 31 months. Two patients (4%) developed small bony cysts around the cup without clinical symptoms. Smeraglia et al. [40] reported no major or minor complications in either group. Tan et al. [42] noted prolonged scar hypersensitivity in two LRTI patients and one dislocated prosthesis cup.

Radiological complications included radiolucent lines around prosthetic components in the studies by Guzzini et al. [41] (cup: 6.94%, stem: 9.72%) and Falkner et al. [44] (small bony cysts in two patients), though none required intervention.

#### 3.5.6. Patient Satisfaction

Three studies [40,41,44] assessed patient satisfaction, with all reporting high satisfaction rates for both procedures. However, two studies [40,41] found slightly higher satisfaction scores in the prosthesis groups.

Guzzini et al. [41] reported satisfaction scores of 9.20/10 for prostheses versus 9.03/10 for LRTI (*p* > 0.05). Smeraglia et al. [40] found that 73.1% of patients rated their prosthesis outcome as “excellent” compared to only 15.4% for LRTI, with the remaining LRTI outcomes rated as “very good” (38.5%) or “good” (46.1%).

#### 3.5.7. Long-Term Follow-Up and Implant-Related Complications

A critical limitation identified across all included studies is the relatively short follow-up duration, with maximum follow-up of 36 months in the longest study [44]. This timeframe is insufficient to fully evaluate long-term implant performance, particularly concerning potential adverse local tissue reactions, implant degradation, and late failures.

Radiological evidence of potential implant concerns was documented in two studies. Guzzini et al. [41] reported radiolucent lines around prosthetic components (cup: 6.94%, stem: 9.72%), though none required intervention during the 24-month follow-up. Falkner et al. [44] identified small bony cysts around the cup in two patients (4%) at 36 months, also without clinical symptoms. One prosthesis dislocation requiring conversion to resection arthroplasty occurred at 31 months in the Falkner study.

Review of the broader literature reveals sporadic case reports and small series documenting late complications with trapeziometacarpal prostheses beyond 3–5 years, including aseptic loosening, osteolysis, metal wear, synovitis, and implant migration [45,46,47,48,49,50,51,52,53,54]. These reports, while not systematically captured in our comparative studies, raise important questions about long-term implant durability, particularly in elderly patients with osteoporotic bone. The absence of long-term comparative data (>5 years) represents a significant evidence gap that limits our ability to make definitive recommendations for elderly patients, who may have reduced bone quality but also lower functional demands and limited life expectancy affecting the clinical significance of late implant failure.

Future research must prioritize extended follow-up periods (minimum 5–10 years) to adequately assess implant longevity, the true incidence of late complications, and the need for revision surgery—factors critically important for informed decision-making in elderly populations.

#### 3.5.8. Random Effects Meta-Analysis

A random effects meta-analysis was performed on the primary outcomes (pain VAS scores and DASH functional scores) at 6 and 12 months postoperatively. The standardized mean difference (SMD) with 95% confidence intervals was calculated for each outcome. For pain VAS at 6 months, the prosthesis group showed superior results (SMD: −0.42; 95% CI: −0.68 to −0.16; *p* = 0.002; I^2^ = 32%). This advantage diminished at 12 months (SMD: −0.18; 95% CI: −0.39 to 0.03; *p* = 0.094; I^2^ = 28%). DASH scores showed similar patterns, with early advantages for prostheses (6-month SMD: −0.39; 95% CI: −0.64 to −0.14; *p* = 0.003) that decreased by 12 months (SMD: −0.21; 95% CI: −0.46 to 0.04; *p* = 0.102).

Given the heterogeneity in study designs, outcome measures, and follow-up durations, combined with the absence of age-stratified data, we opted against performing a formal meta-analysis specifically for elderly patients. The power analysis (Section 2.3) confirmed that even if such data were available, the sample size of elderly patients across studies would be insufficient (n ≈ 95–105) to provide adequately powered estimates. Instead, we present a qualitative synthesis with careful consideration of how findings from mixed-age populations may apply to elderly patients, acknowledging this as a limitation.

### 3.6. Certainty of Evidence

The certainty of evidence was rated as follows:

- Pain outcomes (VAS): MODERATE certainty (downgraded for indirectness due to mixed-age populations and lack of elderly-specific data)
- Functional outcomes (DASH): MODERATE certainty (downgraded for indirectness)
- Strength outcomes: MODERATE certainty (downgraded for indirectness)
- Complications: LOW certainty (downgraded for indirectness and imprecision due to rare events)
- Long-term outcomes: VERY LOW certainty (downgraded for indirectness, imprecision, and short follow-up duration)

All outcomes were further subject to serious indirectness given the absence of studies designed specifically for patients ≥70 years old.

## 4. Discussion

This systematic review represents the first comprehensive attempt to synthesize evidence comparing trapeziectomy with LRTI versus dual-mobility prosthesis specifically for elderly patients over 70 years old with thumb CMC arthritis. However, our findings reveal a critical gap in the literature: no studies have been designed specifically for this population, despite its rapidly growing size and unique clinical needs. While elderly patients represented 25–41% of participants in the five included comparative studies, the absence of age-stratified analyses limits our ability to draw definitive conclusions for this vulnerable group. Nevertheless, the available evidence, interpreted with appropriate caution, provides valuable preliminary insights that can inform clinical decision-making while highlighting the urgent need for dedicated elderly-focused research.

The methodological limitations of this review must be explicitly acknowledged. First, our analysis encompasses mixed-age populations (mean ages 57–68.2 years) rather than exclusively elderly patients. While we attempted to obtain age-stratified data from all study authors, none had performed such subanalyses. Second, the observed gender disparity (77–90% female in three studies) reflects the true epidemiological pattern of CMC arthritis but may limit generalizability to male elderly patients. Third, the inclusion of one small study (13 patients) [42] was necessary given the paucity of comparative evidence but adds heterogeneity. Fourth, maximum follow-up of 36 months is insufficient to evaluate long-term implant performance, particularly concerning for elderly patients with compromised bone quality. Fifth, our power analysis confirmed that even pooled data provide inadequate sample size for robust age-stratified conclusions. Despite these limitations, we contend that providing carefully interpreted evidence from studies that include elderly patients is more valuable than offering no guidance, particularly given the clinical urgency of treating CMC arthritis in this growing population.

Our findings demonstrate that both procedures effectively alleviate pain and improve function, but with distinct temporal recovery patterns and specific advantages.

A systematic review was not feasible due to heterogeneity in outcome reporting and follow-up times. However, the qualitative synthesis provides valuable insights into the comparative effectiveness of these procedures.

The most consistent finding across all studies was the accelerated recovery associated with dual-mobility prostheses. Patients who received prostheses experienced faster pain relief, earlier functional improvement, and more rapid return to activities of daily living compared to those who underwent trapeziectomy with LRTI [20,40,41,42,43,44,45,46,47,48,49,50,51,52,53,54,55,56,57,58,59]. This advantage was particularly evident in the first 3–6 months postoperatively [20], which is especially relevant for elderly patients who may have a limited life expectancy and for whom rapid functional recovery is paramount. This observation aligns with findings from other comparative studies, though none were specifically designed for the elderly population.

The faster recovery provided by prostheses makes them particularly advantageous for older patients who need to regain independence quickly and resume essential daily activities without prolonged rehabilitation periods [40,41,42,43,44,45,46,53,56].

Grip and pinch strength outcomes were consistently superior in the prosthesis group across all studies, with this advantage was maintained even at final follow-up [40,41,42,43,44,45,46,47,48,49,50,51,52,53,54,55,56,57,58,59]. This finding is particularly significant for elderly patients, as grip strength is not only a measure of hand function but also serves as an important indicator of overall health status, functional independence, and mortality risk in this population. While no study specifically analyzed the elderly subgroup separately, the consistency of this finding across all age groups suggests it likely applies to elderly patients as well.

For elderly individuals who rely on strong grip and pinch strength for basic self-care activities, such as dressing, eating, and using assistive devices, the superior strength outcomes achieved with prostheses may translate to improved quality of life and independence.

Metacarpophalangeal joint stability, a key factor in thumb function, was better preserved with prosthetic replacement, according to Degeorge et al. [43,53]. This suggests that dual-mobility prostheses may be particularly beneficial for elderly patients with concomitant MCP hyperextension, a common finding in advanced CMC arthritis, as they can address both conditions simultaneously without requiring additional procedures. This mechanical advantage may be particularly relevant for elderly patients, who often present with more advanced disease and associated deformities.

The stabilization of the thumb column by prostheses reduces secondary complications such as hyperextension deformities, which weaken pinch mechanics, potentially making prostheses a better choice for elderly patients with pre-existing MCP instability.

Despite these advantages, some important considerations must be addressed when recommending dual-mobility prostheses for elderly patients. While complication rates were comparable between the two procedures across studies, the nature of the complications differed. LRTI complications were typically minor and self-limiting (e.g., trigger thumb or paresthesia) [60,61], while prosthesis complications, though rare, could be more serious (e.g., dislocation or aseptic loosening) [47,50,53] and potentially require revision surgery.

This is particularly relevant for elderly patients who may face increased surgical risk due to comorbidities.

For elderly patients with significant comorbidities or poor bone quality, trapeziectomy with LRTI may be the safer choice due to its lower surgical complexity, shorter operative time, and fewer risks of implant-related complications. Moreover, LRTI provides reliable pain relief and functional outcomes, making it a dependable option for elderly individuals with low functional demands who prioritize durability over immediate strength gains.

The intra-patient comparison by Smeraglia et al. [40] provides particularly compelling evidence, as it controls for individual variables such as pain tolerance, occupation, and baseline function. Their finding that patients consistently preferred the prosthesis-treated hand over the LRTI-treated hand adds weight to the subjective benefits of prosthetic replacement [55,56]. However, this study included only 26 patients, with only 41% being over 70 years old, limiting its generalizability to the elderly population.

This preference highlights the greater satisfaction among patients with prostheses, though whether this preference holds true specifically in the elderly subgroup remains uncertain due to the lack of age-stratified analyses.

For elderly patients specifically, several factors should influence the decision-making process:

- Their typically lower functional demands and limited life expectancy might mitigate concerns about long-term prosthesis survival.
- The faster recovery associated with prostheses may be especially valuable in maintaining independence and quality of life.
- The superior grip and pinch strength outcomes with prostheses may help elderly patients maintain functional independence longer, potentially delaying institutionalization.

However, certain characteristics of elderly patients might favor LRTI, including the following:

- Poor bone quality (which may compromise prosthesis fixation).
- Significant comorbidities that increase surgical risk (as LRTI generally requires a shorter operative time).
- Very limited functional demands where the slight strength advantage of prostheses would be inconsequential.

Trapeziectomy with LRTI may be better suited for elderly patients with limited functional needs or those who are frail, as it avoids the risks associated with implant loosening and provides satisfactory pain relief and stability.

Future studies should include longer follow-up periods to evaluate prosthesis durability, stratified analyses by age groups, and cost-effectiveness evaluations. Dedicated trials focusing specifically on elderly populations are needed to provide stronger evidence for this growing patient demographic.

While dual-mobility prostheses may entail higher initial costs due to the implants and surgical expertise required, their faster recovery and superior strength outcomes may offset these costs by reducing the need for prolonged rehabilitation and dependence on caregivers, particularly in elderly populations.

### 4.1. Gender Considerations and Study Power Limitations

The marked female predominance in three of our included studies (77–90%) warrants specific discussion. This distribution accurately reflects the epidemiological reality of CMC arthritis, which disproportionately affects postmenopausal women due to hormonal influences on cartilage metabolism, joint laxity, and bone quality [1,2]. However, this gender imbalance means our findings may be most applicable to elderly women, with less certainty regarding outcomes in elderly men. The intersection of advanced age, female sex, and osteoporosis creates a unique clinical scenario that may particularly favor LRTI over prosthetic replacement in some cases, given concerns about implant fixation in poor-quality bone.

The inclusion of the Tan et al. study [42], despite its small sample size (13 patients, 15 thumbs), requires justification. While this study alone is clearly underpowered to draw definitive conclusions, its exclusion would remove approximately 35% of the elderly patient data available in the comparative literature. Given the severe scarcity of evidence, we believe its inclusion in a qualitative synthesis is warranted, though we have weighted our interpretations accordingly and explicitly noted this limitation. Our power analysis confirms that even with all five studies combined, the elderly subgroup sample (n ≈ 95–105) remains inadequate for robust statistical conclusions, underscoring the critical need for larger, dedicated studies.

### 4.2. Evolution of Dual-Mobility Prostheses and Material Considerations

The dual-mobility trapeziometacarpal prosthesis represents a significant evolution from earlier fixed-bearing designs that demonstrated high failure rates in the 1990s and early 2000s. Historical prosthetic failures were primarily attributed to inadequate polyethylene quality, poor implant design leading to stress concentration, and lack of dual-mobility articulation resulting in edge loading and accelerated wear.

Modern dual-mobility designs address these limitations through several key innovations: (1) highly cross-linked polyethylene cups with superior wear resistance, reducing volumetric wear by 80–95% compared to conventional polyethylene; (2) dual articulation surfaces (metacarpal head within the cup, cup within the trapezial component) that distribute forces more physiologically and reduce range-of-motion constraints; (3) hydroxyapatite-coated or porous-coated stems promoting biological fixation in osteoporotic bone; and (4) modular component designs allowing intraoperative customization to individual anatomy.

Despite these advances, case reports continue to document late complications including aseptic loosening (typically 4–8 years post-implantation), periprosthetic osteolysis secondary to polyethylene or metal debris, cup migration requiring revision, and synovitis from wear particles. These reports, while representing individual cases rather than systematic series, raise important concerns about cumulative long-term risk—particularly relevant for elderly patients with compromised bone quality who may lack the physiological reserve to tolerate revision surgery.

The superiority of dual-mobility prostheses observed in our review regarding strength preservation and early functional recovery must therefore be weighed against this uncertain long-term complication profile. For elderly patients specifically, the clinical significance of superior strength outcomes may diminish if implant-related complications emerge within their remaining lifespan, particularly if such complications compromise the very independence these procedures aim to preserve.

#### Long-Term Implant Performance and Adverse Tissue Reactions: A Critical Evidence Gap

A fundamental concern inadequately addressed by current evidence is the long-term performance of dual-mobility prostheses, particularly regarding implant degradation and adverse local tissue reactions. The maximum 36-month follow-up in our included studies is insufficient to evaluate these critical outcomes, which often manifest years after implantation.

While our systematic comparison studies reported minimal complications, the broader literature contains concerning case reports and small series documenting late prosthetic failures including aseptic loosening (reported 4–8 years post-implantation), periprosthetic osteolysis, wear-induced synovitis, metal-on-polyethylene debris reactions, and cup migration requiring revision [47,50,52,53,54]. These complications, though individually rare, raise important questions about cumulative risk over extended timeframes.

For elderly patients specifically, several factors complicate risk-benefit assessment:

1. Reduced bone quality may compromise implant fixation and accelerate periprosthetic osteolysis
2. Lower functional demands may reduce wear rates but also decrease the relative benefit of superior strength outcomes
3. Limited life expectancy may mean patients do not live long enough to experience late implant failure
4. Reduced physiological reserve and increased comorbidities make revision surgery particularly high-risk if implant failure occurs
5. Potential for subclinical adverse tissue reactions (e.g., metallosis, chronic synovitis) that may not cause symptoms requiring revision but could affect quality of life

The absence of systematic long-term surveillance (>5 years) of elderly patients specifically represents perhaps the most critical gap in current evidence. Future studies must include:

- Minimum 5–10-year follow-up periods
- Standardized protocols for detecting subclinical implant complications (advanced imaging, biomarkers)
- Age-stratified analyses to determine whether elderly patients experience different complication profiles
- Cost-effectiveness analyses incorporating revision surgery risk
- Patient-reported outcomes specifically validated for elderly populations

Until such evidence is available, prosthetic replacement in elderly patients should be approached with appropriate caution, particularly in those over 75 years or with significant osteoporosis, where LRTI may offer a safer, albeit slower-recovering, alternative.

All key consideration and findings for treatment selection in elderly patients are summarized in Table 2 and Table 3.

## 5. Limitations

The limitations of this review include the following:

- The absence of studies designed specifically for elderly patients over 70 years old, requiring extrapolation from mixed-age populations
- Lack of age-stratified subgroup analyses in any included study, despite multiple author contacts
- Marked gender imbalance (77–90% female in three studies) limiting generalizability to elderly men
- Power analysis confirming inadequate sample size for robust age-specific conclusions even with pooled data
- The heterogeneity of outcome measures and follow-up periods across studies.
- Inclusion of one substantially underpowered study (n = 13) necessitated by severe evidence scarcity
- The relatively short follow-up periods (maximum 36 months) that preclude evaluation of long-term implant performance, adverse tissue reactions, and late failures—particularly critical for elderly patients with compromised bone quality.
- The small number of included studies (n = 5), due to the strict inclusion criteria.
- The lack of cost-effectiveness analyses in any of the included studies.
- Inability to account for important elderly-specific factors such as comorbidity burden, frailty status, cognitive function, and social support—all of which may influence treatment selection and outcomes
- Absence of standardized protocols for detecting subclinical implant complications in any included study

## 6. Conclusions

This systematic review reveals a critical evidence gap: despite representing the population most affected by thumb CMC arthritis, no comparative studies have been designed specifically for patients over 70 years old. Our analysis of five studies that included elderly patients (25–41% of populations) provides preliminary insights but cannot substitute for dedicated elderly-focused research.

Based on mixed-age populations including elderly patients, both procedures effectively treat CMC arthritis with distinct temporal and functional profiles (Table 4):

- Dual-mobility prostheses offer faster pain relief (1–3 months vs. 6–12 months), superior strength outcomes maintained throughout follow-up, and better preservation of thumb architecture—potentially valuable for relatively healthy elderly patients (70–75 years) with good bone quality and high functional demands.
- Trapeziectomy with LRTI provides reliable long-term outcomes with proven durability, minor self-limiting complications, and no implant-related concerns—potentially safer for frail elderly patients (>75 years), those with poor bone quality, or when revision surgery would be prohibitive.

Critical Limitations Precluding Definitive Elderly-Specific Recommendations:

- No age-stratified analyses available; insufficient male representation (77–90% female in 3/5 studies)
- Maximum 36-month follow-up inadequate for assessing long-term implant performance in elderly bone
- Power analysis confirms inadequate sample size for robust elderly-specific conclusions
- Unknown long-term rates of prosthetic loosening, osteolysis, and revision in elderly populations

Clinical Decision-Making Framework: Treatment selection should be individualized considering: patient age and frailty status; bone quality and comorbidity burden; functional demands and life expectancy; patient preferences for rapid recovery vs. long-term proven reliability; surgical expertise and institutional capabilities; and capacity to undergo potential revision surgery.

Essential Future Research Priorities: Prospective comparative trials exclusively in patients ≥70 years with balanced gender representation, minimum 5–10-year follow-up evaluating implant longevity and late complications, age-stratified analyses of existing cohorts, systematic surveillance for subclinical adverse tissue reactions in elderly bone, and cost-effectiveness analyses incorporating quality-adjusted life years and revision surgery risk.

Treatment selection should consider patient age, bone quality, functional demands, comorbidity burden, and surgeon expertise. Based on mixed-age evidence with 25–41% elderly representation, dual-mobility prostheses may offer advantages for relatively healthy patients (70–75 years) with good bone quality and high functional demands, while trapeziectomy with LRTI may be preferable for patients >75 years, those with significant osteoporosis, or when revision surgery would be prohibitive. However, these considerations remain provisional pending dedicated elderly-focused studies with age-stratified analyses.

## Figures and Tables

**Table 1:** Selected studies. Note: Where entries show ‘X patients (Y thumbs)’, this indicates X patients with Y thumbs treated, accounting for bilateral cases.

Author	Type of Prosthesis	Type of LRTI	Type of Study	Total Patients (Thumbs)	Allocation	Sex		Mean Age	Mean Follow-Up
					Prosthesis/LRTI	M	F		
Smeraglia F., 2025 [40]	Touch Maia	Altissimi	Retrospective cohort	26 (52 thumbs)	26/26	3	23	61.23 (45–79)	24 months
Guzzini M., 2024 [41]	Touch	Robinson	Randomized Controlled Trial	136 (147 thumbs)	65/71	25	111	67.21 (52–79)	24 months
Tan TH., 2024 [42]	Touch	Weilby	Retrospective cohort	13 (15 thumbs)	7/6	5	8	62.5 (53–75)	14 months
Degeorge B., 2018 [43]	Maia	Altissimi	Retrospective cohort	69 (69 thumbs)	41/28	7	62	63.2 (42–79)	20 months
Falkner F., 2024 [44]	Touch	Weilby	Prospective cohort	69 (71 thumbs)	49/22	38	14	57 (41–76)	36 months

**Table 2:** Comparative Summary: Key Considerations for Treatment Selection in Elderly Patients.

Domain	Dual-Mobility Prosthesis	Trapeziectomy with LRTI
Early recovery (0–6 months)	Superior pain relief at 1–3 months Faster functional improvement Earlier return to ADLs	Slower pain relief plateau at 6–12 months Gradual functional gains Extended rehabilitation period
Strength outcomes	Superior grip strength (maintained throughout follow-up) Better pinch strength (2.7 kg advantage at 3 years) Preserved first ray length	Gradual strength improvement First ray shortening (mean 8.5 mm) Comparable final grip, inferior pinch
Mechanical advantages	Preserves thumb length Stabilizes MCP hyperextension Maintains carpal height	Progressive first ray shortening May not address MCP instability Requires additional procedures for MCP
Complications	Rare but potentially serious (dislocation 2%, loosening) May require revision surgery Radiolucent lines 7–10% (mostly asymptomatic)	More frequent but minor (trigger thumb, paresthesia) Generally self-limiting Rarely require intervention
Long-term concerns	Limited data beyond 3 years Potential for late loosening/osteolysis Wear debris reactions uncertain Revision surgery complex	Decades of long-term data Stable pain relief maintained No prosthetic implant; biological tissue integration without foreign material No revision risk
Ideal elderly candidate	Age 70–75 years Good bone quality High functional demands Medically fit for potential revision	Age > 75 or frail Poor bone quality/osteoporosis Limited functional demands Multiple comorbidities
Special considerations	Requires surgical expertise Higher initial cost Rapid independence restoration Addresses concurrent MCP instability	Technically simpler Lower cost Proven reliability Suitable when expertise limited

**Table 3:** Key Findings Summary.

Key Findings Summary
***Evidence Quality and Limitations***
No studies designed exclusively for patients ≥70 years
Elderly patients represent 25–41% of included populations (n ≈ 95–105 total)
No age-stratified analyses available despite author contacts
Gender imbalance (77–90% female in 3/5 studies) limits male applicability
Maximum follow-up 36 months—insufficient for long-term implant assessment
Power analysis confirms inadequate sample size for robust elderly-specific conclusions
Comparative Outcomes (Mixed-Age Populations Including Elderly)
***Early Recovery (0–6 months)***
Dual-mobility prosthesis: Superior pain relief (VAS difference 1.9 points at 3 months, *p* < 0.0001), faster functional improvement (DASH < 35 in >95% at 6 months vs. 12 months for LRTI)
LRTI: Slower but progressive improvement, plateau at 6–12 months
***Strength Outcomes (Maintained Throughout Follow-up)***
Dual-mobility prosthesis: Superior grip and pinch strength (key pinch 2.7 kg advantage at 3 years, *p* < 0.0001), preserved first ray length
LRTI: First ray shortening (mean 8.5 mm in 66.7%), comparable final grip but inferior pinch
***Complications***
Dual-mobility prosthesis: Rare (dislocation 2%, radiolucent lines 7–10%) but potentially serious, may require revision
LRTI: More frequent (22.6%) but minor and self-limiting (trigger thumb, paresthesia)
***Patient Satisfaction***
Dual-mobility prosthesis: 73.1% rated “excellent” vs. 15.4% for LRTI (intra-patient comparison)
Both procedures: High overall satisfaction (>9/10)

**Table 4:** **Provisional Treatment Considerations Based on Mixed-Age Evidence**. This table presents provisional considerations extrapolated from mixed-age populations. These are NOT evidence-based recommendations for elderly patients specifically. Terms like “ideal candidate” reflect clinical reasoning, not data-driven conclusions. Definitive guidelines require dedicated studies in patients ≥70 years with age-stratified analyses and minimum 5–10-year follow-up.

Patient Characteristic	Favor Dual-Mobility Prosthesis	Favor LRTI
Age & general health	70–75 years, medically fit, non-frail	>75 years, frail, multiple comorbidities
Bone quality	Good cortical thickness, minimal osteoporosis	Osteoporotic, poor bone quality
Functional demands	High (independent living, manual activities)	Limited (sedentary, dependent care)
Life expectancy	>10 years	<5 years
MCP joint status	Hyperextension present	Stable or minimal instability
Priority	Rapid functional recovery, strength preservation	Proven reliability, minimal revision risk
Revision tolerance	Medically able to undergo revision if needed	High surgical risk, revision prohibitive
Surgical expertise	Available prosthetic expertise	Limited expertise or resources

## Data Availability

The data presented in this study are available on request from the corresponding author.

## Supplementary Materials

The following supporting information can be downloaded at: https://www.mdpi.com/article/10.3390/jcm15031137/s1, Table S1: PRISMA checklist.

## Abbreviations

The following abbreviations are used in this manuscript:

LRTI	Ligament Reconstruction and Tendon Interposition
CMC	Carpometacarpal
VAS	Visual Analog Scale
DASH	Disability of the Arm, Shoulder, and Hand
RCTs	Randomized Controlled Trials
MCP	Metacarpophalangeal
CI	Confidence Interval

## References

[1] van der Oest M.J.W., Duraku L.S., Andrinopoulou E.R., Wouters R., Bierma-Zeinstra S., Selles R., Zuidam J. (2021). The prevalence of radiographic thumb base osteoarthritis: A meta-analysis. Osteoarthr. Cartil..

[2] Østerås N., Kjeken I., Smedslund G., Moe R.H., Slatkowsky-Christensen B., Uhlig T., Hagen K.B. (2017). Exercise for hand osteoarthritis. Cochrane Database Syst. Rev..

[3] Passiatore M., Taccardo G., Cilli V., Rovere G., Liuzza F., Pannuto L., De Vitis R. (2023). Surgical treatment of carpometacarpal thumb arthritis with trapeziectomy and intra-tendon (FCR) suspension with one-loop APL: Comparative cohort study. BMC Musculoskelet. Disord..

[4] Taccardo G., De Vitis R., Parrone G., Milano G., Fanfani F. (2014). Surgical treatment of trapeziometacarpal joint osteoarthritis. Joints.

[5] Day W., Rancu A., Halim A., Gouzoulis M.J., Joo P.Y., Grauer J.N. (2025). National Trends of Surgical Interventions for Thumb Carpometacarpal Arthritis from 2010 to 2022. J. Hand Surg. Am..

[6] Chen K., Shun Y., Xiang W. (2023). Differences between trapeziometacarpal arthrodesis and trapeziectomy with ligament reconstruction for the treatment of trapeziometacarpal osteoarthritis: A systematic review and meta-analysis. Acta Orthop. Belg..

[7] Kim C.H., Lee D.H., Lee J.S., Jung H.S. (2025). Arthrodesis Versus Ligament Reconstruction and Tendon Interposition for Thumb Carpometacarpal Joint Arthritis: A Systematic Review and Meta-Analysis. J. Hand Surg. Am..

[8] Hippensteel K.J., Calfee R., Dardas A.Z., Gelberman R., Osei D., Wall L. (2017). Functional Outcomes of Thumb Trapeziometacarpal Arthrodesis with a Locked Plate Versus Ligament Reconstruction and Tendon Interposition. J. Hand Surg. Am..

[9] Robles-Molina M.J., López-Caba F., Gómez-Sánchez R.C., Cárdenas-Grande E., Pajares-López M., Hernández-Cortés P. (2017). Trapeziectomy with Ligament Reconstruction and Tendon Interposition Versus a Trapeziometacarpal Prosthesis for the Treatment of Thumb Basal Joint Osteoarthritis. Orthopedics.

[10] De Smet L., Vandenberghe L., Degreef I. (2013). Long-term outcome of trapeziectomy with ligament reconstruction and tendon interposition (LRTI) versus prosthesis arthroplasty for basal joint osteoarthritis of the thumb. Acta Orthop. Belg..

[11] Qureshi M.K., Halim U.A., Khaled A.S., Roche S.J., Arshad M.S. (2021). Trapeziectomy with Ligament Reconstruction and Tendon Interposition versus Trapeziometacarpal Joint Replacement for Thumb Carpometacarpal Osteoarthritis: A Systematic Review and Meta-Analysis. J. Wrist Surg..

[12] Froschauer S.M., Holzbauer M., Hager D., Schnelzer R., Kwasny O., Duscher D. (2020). Elektra prosthesis versus resection-suspension arthroplasty for thumb carpometacarpal osteoarthritis: A long-term cohort study. J. Hand Surg. Eur. Vol..

[13] Teissier J., Teissier P., Toffoli A. (2021). Trapeziometacarpal prostheses. Hand Surg. Rehabil..

[14] Maling L., Rooney A. (2025). Outcomes of dual-mobility trapeziometacarpal arthroplasties: A systematic review. J. Hand Surg. Eur. Vol..

[15] Newton A., Talwalkar S. (2022). Arthroplasty in thumb trapeziometacarpal (CMC joint) osteoarthritis: An alternative to excision arthroplasty. J. Orthop..

[16] Schindele S.F., Marks M., Beaud X., Reischenböck V., Herren D.B. (2025). Correction of metacarpophalangeal joint hyperextension following trapeziometacarpal joint implant arthroplasty: A case-control study. J. Hand Surg. Eur. Vol..

[17] Herren D.B., Ishikawa H., Rizzo M., Ross M., Solomons M. (2022). Arthroplasty in the hand: What works and what doesn’t?. J. Hand Surg. Eur. Vol..

[18] Villari E., Langone L., Pilla F., Chiaramonte I., Ramponi L., Faldini C. (2025). Dual mobility trapeziometacarpal prosthesis: A review of the current literature. Hand Surg. Rehabil..

[19] Latelise B., Ben Brahim E., Prasil L., Freslon M. (2024). Complications of prosthesis versus trapeziectomy in trapeziometacarpal joint arthritis: A systematic review. Hand Surg. Rehabil..

[20] Herren D.B., Marks M., Neumeister S., Schindele S. (2023). Short-term recovery after implant versus resection arthroplasty in trapeziometacarpal joint osteoarthritis. J. Hand Surg. Eur. Vol..

[21] Bohannon R.W. (2019). Grip Strength: An Indispensable Biomarker for Older Adults. Clin. Interv. Aging.

[22] Mehmet H., Yang A.W.H., Robinson S.R. (2020). Measurement of hand grip strength in the elderly: A scoping review with recommendations. J. Bodyw. Mov. Ther..

[23] Lee S.C., Wu L.C., Chiang S.L., Lu L.-H., Chen C.-Y., Lin C.-H., Ni C.-H., Lin C.-H. (2020). Validating the Capability for Measuring Age-Related Changes in Grip-Force Strength Using a Digital Hand-Held Dynamometer in Healthy Young and Elderly Adults. BioMed Res. Int..

[24] Porto J.M., Nakaishi A.P.M., Cangussu-Oliveira L.M., Freire Júnior R.C., Spilla S.B., Abreu D.C.C. (2019). Relationship between grip strength and global muscle strength in community-dwelling older people. Arch Gerontol. Geriatr..

[25] Rezaei A., Bhat S.G., Cheng C.H., Pignolo R.J., Lu L., Kaufman K.R. (2024). Age-related changes in gait, balance, and strength parameters: A cross-sectional study. PLoS ONE.

[26] Shinya Y., Ikeguchi R., Noguchi T., Ando M., Yoshimoto K., Sakamoto D., Iwai T., Matsuda S. (2023). Radiographic Evaluation after Arthroscopic Partial Trapeziectomy with Suture-button Suspensionplasty for Thumb Carpometacarpal Arthritis. Plast. Reconstr. Surg. Glob. Open.

[27] Downing N.D., Davis T.R. (2001). Trapezial space height after trapeziectomy: Mechanism of formation and benefits. J. Hand Surg. Am..

[28] Duché R., Trabelsi A. (2022). The concept of first metacarpal M1-M2 arch. New interest in trapeziometacarpal prostheses. Hand Surg. Rehabil..

[29] Salas C., Mercer D.M., O’Mahony G., Love J., LaBaze D., Moneim M.S. (2016). Thumb Metacarpal Subsidence After Partial Trapeziectomy with Capsular Interposition Arthroplasty: A Biomechanical Study. Hand.

[30] Giacalone F., di Summa P.G., Fenoglio A., Sard A., Dutto E., Ferrero M., Bertolini M., Garcia-Elias M. (2017). Resurfacing Capitate Pyrocarbon Implant versus Proximal Row Carpectomy Alone: A Comparative Study to Evaluate the Role of Capitate Prosthetic Resurfacing in Advanced Carpal Collapse. Plast. Reconstr. Surg..

[31] De Vitis R., Passiatore M., Cilli V., Pamelin E., Velluto C., Ceravolo I., D’oRio M., Ferrari F., Taccardo G. (2021). Secondary Wrist Arthritis in Active Workers: Does Capitate Pyrocarbon Resurfacing (RCPI) Improve Proximal Row Carpectomy? A Retrospective Cohort Study. J. Hand Surg. Asian Pac. Vol..

[32] Paul A.W., Athens C.M., Patel R., Rizzo M., Rhee P.C. (2022). Effect of Trapeziectomy on Carpal Stability. Hand.

[33] Rocchi L., De Vitis R., Pietramala S., Fulchignoni C., D’Orio M., Mazzone V., Marcuzzi A. (2022). Resurfacing Capitate Pyrocarbon Implant for the treatment of advanced wrist arthritis in the elderly: A retrospective study. Eur. Rev. Med. Pharmacol. Sci..

[34] Cannella A., Sassara G.M., Caruso L., Rapisarda A.M., Passiatore M., Cilli V., Guzzini M., De Vitis R. (2025). Indication for Radial or Carpal Resurfacing for Wrist Arthritis in Elderly Patients (over 70): A Systematic Review of the Literature. J. Clin. Med..

[35] Umehara T., Kaneguchi A., Kawakami W., Katayama N., Kito N. (2022). Association of muscle mass and quality with hand grip strength in elderly patients with heart failure. Heart Vessels.

[36] Norman K., Stobäus N., Gonzalez M.C., Schulzke J.D., Pirlich M. (2011). Hand grip strength: Outcome predictor and marker of nutritional status. Clin. Nutr..

[37] Bohannon R.W. (2008). Hand-grip dynamometry predicts future outcomes in aging adults. J. Geriatr. Phys. Ther..

[38] Beseler M.R., Rubio C., Duarte E., David H., Cruz G.M., Manuel G., Enrique V. (2014). Clinical effectiveness of grip strength in predicting ambulation of elderly inpatients. Clin. Interv. Aging.

[39] Cai Y., Liu L., Wang J., Gao Y., Guo Z., Ping Z. (2021). Linear association between grip strength and all-cause mortality among the elderly: Results from the SHARE study. Aging Clin. Exp. Res..

[40] Smeraglia F., Carità E., Frittella G., Tamborini F., Diaz L., Donadelli A., Guzzini M. (2025). Intra-patient comparison of trapeziectomy with LRTI and dual mobility prosthesis for trapeziometacarpal osteoarthritis: A multicenter observational study. Eur. J. Orthop. Surg. Traumatol..

[41] Guzzini M., Arioli L., Annibaldi A., Pecchia S., Latini F., Ferretti A. (2024). Interposition Arthroplasty versus Dual Cup Mobility Prosthesis in Treatment of Trapeziometacarpal Joint Osteoarthritis: A Prospective Randomized Study. Hand.

[42] Tan T.H., Kang H.Y.G. (2024). Comparative Study of Trapeziectomy with Weilby Suspensionplasty versus Implant Arthroplasty for Thumb Carpometacarpal Joint Arthritis in an Asian Population. J. Hand Surg. Asian Pac. Vol..

[43] Degeorge B., Dagneaux L., Andrin J., Lazerges C., Coulet B., Chammas M. (2018). Do trapeziometacarpal prosthesis provide better metacarpophalangeal stability than trapeziectomy and ligamentoplasty?. Orthop. Traumatol. Surg. Res..

[44] Falkner F., Tümkaya A.M., Thomas B., Böcker A., Aman M., Bickert B., Harhaus L., Panzram B. (2024). Resection arthroplasty versus dual mobility prosthesis in the treatment of trapeziometacarpal joint osteoarthritis: A 3 year non-randomized prospective study. J. Orthop..

[45] Picchi A., Rovere G., Fulchignoni C., Bosco F., Venosa M., Andriollo L., De Vitis R., Smakaj A., Fidanza A. (2025). Dual Mobility Arthroplasty Versus Suspension Tenoplasty for Treatment of Trapezio–Metacarpal Joint Arthritis: A Clinical Trial. Appl. Sci..

[46] Piccirilli E., di Sette P., Rampoldi M., Primavera M., Salvati C., Tarantino U. (2024). Comparative Analysis of Prosthetic (Touch) and Arthroplastic Surgeries for Trapeziometacarpal Arthrosis: Functional Outcomes and Patient Satisfaction With a 2-Year Follow-Up. J. Hand Surg. Glob. Online.

[47] Klim S.M., Glehr R., Graef A., Amerstorfer F., Leithner A., Glehr M. (2023). Total joint arthroplasty versus resection-interposition arthroplasty for thumb carpometacarpal arthritis: A randomized controlled trial. Acta Orthop..

[48] Martínez-Martínez F., García-Hortelano S., García-Paños J.P., Moreno-Fernández J.M., Martín-Ferrero M.Á. (2016). Estudio clínico comparativo de 2 técnicas quirúrgicas de rizartrosis del pulgar [Comparative clinical study of 2 surgical techniques for trapeziometacarpal osteoarthritis]. Rev. Esp. Cir. Ortop. Traumatol..

[49] Catalano L., Horne L.T., Fischer E., Barron O.A., Glickel S.Z. (2008). Comparison of ligament reconstruction tendon interposition and trapeziometacarpal interposition arthroplasty for basal joint arthritis. Orthopedics.

[50] De Smet L., Sioen W. (2007). Basal joint osteoarthritis of the thumb: Trapeziectomy, with or without tendon interposition, or total joint arthroplasty? A prospective study. Eur. J. Orthop. Surg. Traumatol..

[51] Fulchignoni C., Pietramala S., Arioli L., Gerace E., De Mauro D., Frittella G., Di Dio E., Grauso M., Merendi G., Rocchi L. (2025). Role of Post-Operative Rehabilitation in TM Joint Arthritis: Functional Outcomes of Interposition Trapeziectomy vs. Prosthesis. J. Funct. Morphol. Kinesiol..

[52] Remy S., Detrembleur C., Libouton X., Bonnelance M., Barbier O. (2020). Trapeziometacarpal prosthesis: An updated systematic review. Hand Surg. Rehabil..

[53] Thorkildsen R.D., Røkkum M. (2019). Trapeziectomy with LRTI or joint replacement for CMC1 arthritis, a randomised controlled trial. J. Plast. Surg. Hand Surg..

[54] Seth I., Bulloch G., Seth N., Fogg Q., Hunter-Smith D.J., Rozen W.M. (2024). Efficacy and Safety of Different Trapezium Implants for Trapeziometacarpal Joint Osteoarthritis: A Systematic Review and Meta-Analysis. Hand.

[55] Nietlispach V., Marks M., Imhof J., Pudic T., Herren D.B. (2025). Which would you choose again? Comparison of trapeziometacarpal implant versus resection arthroplasty in the same patient. J. Hand Surg. Eur. Vol..

[56] Jager T., Barbary S., Dap F., Dautel G. (2013). Analyse de la douleur postopératoire et des résultats fonctionnels précoces dans le traitement de la rhizarthrose. Étude prospective comparative de 74 patientes trapézectomie-interposition vs prothèse MAIA^®^ [Evaluation of postoperative pain and early functional results in the treatment of carpometacarpal joint arthritis. Comparative prospective study of trapeziectomy vs. MAIA^®^ prosthesis in 74 female patients]. Chir. Main..

[57] Verhulst K., Dauwe J., Van Nuffel M., De Smet L. (2020). Short-term outcome trapeziectomy with ligament reconstruction and tendon interposition versus trapeziometacarpal prosthesis: A literature review. Acta Orthop. Belg..

[58] Windhofer C.M., Neureiter J., Schauer J., Zimmermann G., Hirnsperger C. (2023). Trapeziectomy versus Maïa Prosthesis in Trapeziometacarpal Osteoarthritis. J. Wrist Surg..

[59] Simón-Pérez C., Frutos-Reoyo E.J., Martín-Ferrero M.Á., Aguado-Maestro I., Guirao-Cano L., Martínez-Martínez F. (2025). Total Arthroplasty Versus Trapeziectomy with Ligamentoplasty for Trapeziometacarpal Osteoarthritis: 5-year Outcomes. Clin. Orthop. Relat. Res..

[60] Herren D.B., Boeckstyns M., Chung K.C., Farnebo S., Hagert E., Tang J.B., Verstreken F., Marks M., the FaiTh Study Group (2024). Diagnostic and treatment recommendations for recurrent or persistent symptoms after trapeziectomy: A Delphi study. J. Hand Surg. Eur. Vol..

[61] De Vitis R., Taccardo G., Passiatore M., Boekstyns M., Marks M., Herren D.B., FaiTh study group (2025). Re: Herren DB, Boeckstyns M, Chung KC et al. Diagnostic and treatment recommendations for recurrent or persistent symptoms after trapeziectomy: A Delphi study. J Hand Surg Eur Vol. 2024. doi: 10.1177/17531934241227386. J. Hand Surg. Eur. Vol..

