# Supplementary material for: Trapeziectomy with LRTI or Dual-Mobility Prosthesis for Thumb Carpometacarpal Arthritis: A Systematic Review with Considerations for Elderly Patients over 70 Years of Age
Source: J Clin Med. 2026 Feb 1;15(3):1137. doi: 10.3390/jcm15031137 (PMC12898151; doi:10.3390/jcm15031137)
Supplement: Supplementary file 1 [file jcm-15-01137-s001.zip › jcm-4105172-supplementary.pdf]

PRISMA 2020 Checklist

Section and Topic	Item #	Checklist item	Location where item is reported
**TITLE**			
Title	1	Identify the report as a systematic review.	1
**ABSTRACT**			
Abstract	2	See the PRISMA 2020 for Abstracts checklist.	1
**INTRODUCTION**			
Rationale	3	Describe the rationale for the review in the context of existing knowledge.	Introduction, paragraphs 1-4 (lines discussing CMC arthritis prevalence, surgical options, lack of elderly-specific evidence)
Objectives	4	Provide an explicit statement of the objective(s) or question(s) the review addresses.	Introduction, final paragraph and Abstract ("This systematic review compares trapeziectomy with LRTI versus dual-mobility prosthesis...")
**METHODS**			
Eligibility criteria	5	Specify the inclusion and exclusion criteria for the review and how studies were grouped for the syntheses.	Section 2.2 "Inclusion Criteria and Exclusion Criteria"
Information sources	6	Specify all databases, registers, websites, organisations, reference lists and other sources searched or consulted to identify studies. Specify the date when each source was last searched or consulted.	Section 2.1 "Search Strategy" (PubMed, Scopus, Web of Science, Cochrane Library; searched up to August 2025)

Section and Topic	Item #	Checklist item	Location where item is reported
Search strategy	7	Present the full search strategies for all databases, registers and websites, including any filters and limits used.	Section 2.1 "Search Strategy" (complete search string provided)
Selection process	8	Specify the methods used to decide whether a study met the inclusion criteria of the review, including how many reviewers screened each record and each report retrieved, whether they worked independently, and if applicable, details of automation tools used in the process.	Section 2.3 "Data Extraction and Quality Assessment" (two independent reviewers, third for discrepancies)
Data collection process	9	Specify the methods used to collect data from reports, including how many reviewers collected data from each report, whether they worked independently, any processes for obtaining or confirming data from study investigators, and if applicable, details of automation tools used in the process.	Section 2.3 "Data Extraction and Quality Assessment" (standardized form, two reviewers independently, authors contacted for age-stratified data)
Data items	10a	List and define all outcomes for which data were sought. Specify whether all results that were compatible with each outcome domain in each study were sought (e.g. for all measures, time points, analyses), and if not, the methods used to decide which results to collect.	Section 2.2 (pain reduction VAS, functional improvement DASH, complications, patient satisfaction); Section 2.3 (primary outcomes at specific timepoints)
	10b	List and define all other variables for which data were sought (e.g. participant and intervention characteristics, funding sources). Describe any assumptions made about any missing or unclear information.	Section 2.3 and Table 1 (patient demographics,

PRISMA 2020 Checklist

Section and Topic	Item #	Checklist item	Location where item is reported
			age, sex, follow-up duration)
Study risk of bias assessment	11	Specify the methods used to assess risk of bias in the included studies, including details of the tool(s) used, how many reviewers assessed each study and whether they worked independently, and if applicable, details of automation tools used in the process.	Section 2.3 "Data Extraction and Quality Assessment" (Cochrane tool for RCTs, Newcastle-Ottawa Scale for observational studies, two independent reviewers)
Effect measures	12	Specify for each outcome the effect measure(s) (e.g. risk ratio, mean difference) used in the synthesis or presentation of results.	Section 2.3 (VAS scores, DASH scores); Section 3.5.8 (SMD with 95% CI)
Synthesis methods	13a	Describe the processes used to decide which studies were eligible for each synthesis (e.g. tabulating the study intervention characteristics and comparing against the planned groups for each synthesis (item #5)).	Section 2.2 and Section 3.5.8 (studies with direct comparisons of both techniques)
	13b	Describe any methods required to prepare the data for presentation or synthesis, such as handling of missing summary statistics, or data conversions.	Section 3.5.8 (random effects meta-analysis for primary outcomes)
	13c	Describe any methods used to tabulate or visually display results of individual studies and syntheses.	Tables 1, 2, 3 and Figure 1 (PRISMA flowchart)
	13d	Describe any methods used to synthesize results and provide a rationale for the choice(s). If meta-analysis was performed, describe the model(s), method(s) to identify the presence and extent of statistical heterogeneity, and software package(s) used.	Section 3.5.8 (random effects meta-analysis, SMD calculations, I^2

Section and Topic	Item #	Checklist item	Location where item is reported
			heterogeneity); also noted that formal meta-analysis for elderly subgroup was not performed due to lack of age-stratified data
	13e	Describe any methods used to explore possible causes of heterogeneity among study results (e.g. subgroup analysis, meta-regression).	Section 3.5.8 (I^2 statistics reported); heterogeneity discussed in Sections 2.2bis and 5 (Limitations)
	13f	Describe any sensitivity analyses conducted to assess robustness of the synthesized results.	Section 2.3 (post-hoc power analysis conducted)
Reporting bias assessment	14	Describe any methods used to assess risk of bias due to missing results in a synthesis (arising from reporting biases).	Section 3.3 "Risk of Bias Assessment" and Section 5 "Limitations" (discussion of publication bias and missing age-stratified data)
Certainty assessment	15	Describe any methods used to assess certainty (or confidence) in the body of evidence for an outcome.	Section 3.3 "Risk of Bias Assessment" (Newcastle-Ottawa Scale scores 6-8/9); extensive discussion in Sections 4.1, 4.2, 4.3 regarding evidence quality and

Section and Topic	Item #	Checklist item	Location where item is reported
			limitations
**RESULTS**			
Study selection	16a	Describe the results of the search and selection process, from the number of records identified in the search to the number of studies included in the review, ideally using a flow diagram.	Section 3.1 "Study Selection" and Figure 1 (PRISMA flowchart)
	16b	Cite studies that might appear to meet the inclusion criteria, but which were excluded, and explain why they were excluded.	Section 2.3 (reasons for exclusion: n=415 single technique, n=237 no elderly patients, n=156 no direct comparison, n=34 insufficient outcomes)
Study characteristics	17	Cite each included study and present its characteristics.	Table 1 and Section 3.2 "Study Characteristics" (all 5 studies cited with characteristics)
Risk of bias in studies	18	Present assessments of risk of bias for each included study.	Section 3.3 "Risk of Bias Assessment"
Results of individual studies	19	For all outcomes, present, for each study: (a) summary statistics for each group (where appropriate) and (b) an effect estimate and its precision (e.g. confidence/credible interval), ideally using structured tables or plots.	Sections 3.4 through 3.5.7 (VAS, DASH, ROM, strength, radiological outcomes, complications, satisfaction - all with specific values and p-values cited for individual studies)
Results of	20a	For each synthesis, briefly summarise the characteristics and risk of bias among contributing studies.	Section 3.2 and

Section and Topic	Item #	Checklist item	Location where item is reported
syntheses			3.3; Section 3.5.8 for meta-analysis results
	20b	Present results of all statistical syntheses conducted. If meta-analysis was done, present for each the summary estimate and its precision (e.g. confidence/credible interval) and measures of statistical heterogeneity. If comparing groups, describe the direction of the effect.	Section 3.5.8 "Random effects meta-analysis" (SMD, 95% CI, p-values, I² values for pain and DASH at 6 and 12 months)
	20c	Present results of all investigations of possible causes of heterogeneity among study results.	Section 3.5.8 (I² statistics); Sections 2.2bis, 3.2bis, 4.1 (discussion of heterogeneity sources)
	20d	Present results of all sensitivity analyses conducted to assess the robustness of the synthesized results.	Section 2.3 (post-hoc power analysis)
Reporting biases	21	Present assessments of risk of bias due to missing results (arising from reporting biases) for each synthesis assessed.	Section 5 "Limitations" (extensive discussion of missing age-stratified data, short follow-up, gender imbalance)
Certainty of evidence	22	Present assessments of certainty (or confidence) in the body of evidence for each outcome assessed.	Section 4 "Discussion," Section 4.3 "Long-term Implant Performance," Section 6 "Conclusion" (extensive discussion of evidence quality and confidence

PRISMA 2020 Checklist

Section and Topic	Item #	Checklist item	Location where item is reported
			limitations)
**DISCUSSION**			
Discussion	23a	Provide a general interpretation of the results in the context of other evidence.	Section 4 "Discussion" (comprehensive interpretation with references to broader literature)
	23b	Discuss any limitations of the evidence included in the review.	Section 5 "Limitations" (detailed discussion of 9 major limitations)
	23c	Discuss any limitations of the review processes used.	Sections 2.2bis, 4, 4.1, and 5 (methodological limitations explicitly addressed)
	23d	Discuss implications of the results for practice, policy, and future research.	Section 6 "Conclusion" (clinical decision-making framework, essential future research priorities); Tables 2 and 3 (treatment selection guidelines)
**OTHER INFORMATION**			
Registration and protocol	24a	Provide registration information for the review, including register name and registration number, or state that the review was not registered.	This review was not prospectively registered
	24b	Indicate where the review protocol can be accessed, or state that a protocol was not prepared.	Section 2.1
	24c	Describe and explain any amendments to information provided at registration or in the protocol.	Section 2.2bis "Rationale for All-Age

PRISMA 2020 Checklist

Section and Topic	Item #	Checklist item	Location where item is reported
			Analysis"
Support	25	Describe sources of financial or non-financial support for the review, and the role of the funders or sponsors in the review.	"Funding: This research received no external funding."
Competing interests	26	Declare any competing interests of review authors.	"Conflicts of Interest: The authors declare no conflicts of interest."
Availability of data, code and other materials	27	Report which of the following are publicly available and where they can be found: template data collection forms; data extracted from included studies; data used for all analyses; analytic code; any other materials used in the review.	"Data Availability Statement: The data presented in this study are available on request from the corresponding author."

From: Page MJ, McKenzie JE, Bossuyt PM, Boutron I, Hoffmann TC, Mulrow CD, et al. The PRISMA 2020 statement: an updated guideline for reporting systematic reviews. BMJ 2021;372:n71. doi: 10.1136/bmj.n71

For more information, visit: http://www.prisma-statement.org/
